# Antidesmoglein 1 and 3 serum IgG and positivity by direct immunofluorescence microscopy is associated with relapse in pemphigus in a prospective bicontinental study

**DOI:** 10.1016/j.jdin.2024.12.004

**Published:** 2025-01-20

**Authors:** Dipankar De, Shipla Shilpa, Sheetanshu Kumar, Kossara Drenovska, Hitaishi Mehta, Nina van Beek, Naresh Sachdeva, Alexandra Fleva, Martin Shahid, Sanjeev Handa, Anastasia Giannakou, Elissaveta Naumova, Rahul Mahajan, Aikaterini Kyriakou, Spaska Lesichkova, Elisabeth Lazaridou, Bishan Dass Radotra, Kamal Kishore, Snejina Vassileva, Aikaterini Patsatsi, Enno Schmidt

**Affiliations:** a Department of Dermatology, Venereology and Leprology, Postgraduate Institute of Medical Education and Research, Chandigarh, India; b Department of Dermatology and Venereology, Faculty of Medicine, Medical University – Sofia, Bulgaria; c Department of Dermatology, University of Lübeck, Lübeck, Germany; d Department of Endocrinology (Immunology Division), Postgraduate Institute of Medical Education and Research, Chandigarh, India; e Department of Immunology-Histocompatibility, Papageorgiou General Hospital, Thessaloniki, Greece; f Department of Clinical Immunology, Faculty of Medicine, Medical University – Sofia, Sofia, Bulgaria; g Dermatology Department, Aristotle University School of Medicine, Papageorgiou General Hospital, Thessaloniki, Greece; h Department of Histopathology, Postgraduate Institute of Medical Education and Research, Chandigarh, India; i Department of Biostatistics, Postgraduate Institute of Medical Education and Research, Chandigarh, India; j Lübeck Institute of Experimental Dermatology, University of Lübeck, Lübeck, Germany

**Keywords:** Autoantibody, desmoglein, remission, pemphigus, prediction of relapse

## Abstract

**Background:**

Prospective studies identifying immunological parameters that can predict clinical relapse in pemphigus are scarce.

**Objective:**

To periodically assess immunological parameters in patients with pemphigus vulgaris and foliaceous in remission to understand immunological events preceding clinical relapse.

**Methods:**

A total of 105 patients were included. Baseline assessment included direct immunofluorescence (DIF), serum IgG against desmoglein (Dsg) 1, IgG, IgG1, and IgG4 against Dsg 3, IgG against the extracellular domains 1 and 2 of Dsg 3, IgG against muscarinic (M3)-AchR, and peripheral CD19+CD27+ memory B cells/plasma cells, repeated every 3 months for up to 12 months or until clinical relapse. DIF was repeated at month 12 and on relapse.

**Results:**

About 29 of 105 patients (28%) experienced a relapse. Longer duration of clinical remission, presence of pruritus and positive anti-Dsg1 at baseline correlated with higher relapse rates. Compared with the visit immediately preceding relapse, a significantly increased number of patients with positive anti-Dsg1 (38% vs 31.1%, *P* = .01), anti-Dsg3 (51.7% vs 41.4%, *P* = .01) and IgG positivity by DIF (85.7% vs 25%, *P* < .001) was observed at the time of relapse.

**Conclusion:**

Regular monitoring of anti-Dsg 1 and anti-Dsg 3 serum levels and DIF positivity during the course of the disease in remission may predict relapse.

**Capsule Summary**

• This study aimed to identify clinical and immunological parameters predicting relapse in pemphigus.
• Pruritus, longer remission duration, and the presence of anti- desmoglein (Dsg)1 IgG at baseline are useful clinical predictors of relapse. Regular monitoring of anti-Dsg 1 and anti-Dsg 3 serum levels and direct immunofluorescence is recommended to predict and manage relapses.

## Introduction

Pemphigus is a group of chronic blistering skin diseases caused by autoantibodies directed against transmembrane glycoproteins expressed on the surface of keratinocytes, resulting in loss of cell-cell adhesion between keratinocytes. The primary subtypes of pemphigus include pemphigus vulgaris (PV) and pemphigus foliaceus (PF), which differ in their clinical presentations and immunopathology. PV, the more common and severe variant, is characterized by mucosal involvement and is mediated by autoantibodies against desmoglein 3 (Dsg3) and, less frequently, Dsg1. In contrast, PF primarily affects the skin without mucosal involvement and is mediated exclusively by anti-Dsg1 autoantibodies.^1^

Pemphigus is the commonest autoimmune bullous disorder in India.^2^^,^^3^ Incidence rates generally range from 0.76 to 5 new cases per million per year.^4^ An incidence rate of 4.7 per million and 8 per million have been reported in Bulgaria and Greece, respectively.^4^ The disease may be potentially prone for relapses after the achievement of clinical remission. In fact, relapse rates varying between 30% and 60% have been reported in randomized controlled trials during or after tapering of the conventional immunosuppressants.^5^^,^^6^ Joly et al^7^ reported relapse in 24% of patients 24 months after rituximab infusion.

As pemphigus shows a relapsing and remitting course, knowledge regarding clinical and serological factors that predict relapse would help clinicians to better manage pemphigus patients and prognosticate their disease course. Previous studies have reported association between relapse and high anti-Dsg1 and anti-Dsg3 titers and direct immunofluorescence (DIF) positivity during remission.^8^, ^9^, ^10^, ^11^

This prospective observational study was performed to serially assess immunological parameters in pemphigus patients (both PV and PF) in complete clinical remission to identify clinical and immunological parameters that can predict relapse.

## Patients and methodology

### Patients

In this prospective multicentric bicontinental study, all patients of PV/PF at clinics of Postgraduate Institute of Medical Education & Research, Chandigarh, Aristotle University of Thessaloniki, Greece, and Medical University of Sofia, Bulgaria, were screened for eligibility from January 2018 to June 2019.^12^ Diagnosis was based on a compatible clinical picture, histopathology of a lesional biopsy and direct immunofluorescence (DIF) of a perilesional biopsy.^13^^,^^14^ At the time of study inclusion, patients were either in complete remission (CR) defined as being lesion-free for at least 2 months while on no (CRoff) or minimal treatment (CRmin).^15^ The study was performed according to the Declaration of Helsinki and written and informed consent were obtained from all patients before inclusion. The study protocol was approved by the respective Institute Ethics Committees in Chandigarh, Thessaloniki, and Sofia.

### Medication used for induction of remission and tapering schemes

Patients had been managed with prednisolone in a dose of 0.5-1 mg/kg/day for induction of remission. Other immunosuppressants utilized for remission included rituximab (*n* *=* 26), mycophenolate mofetil (*n* *=* 4), azathioprine (*n* *=* 35), cyclophosphamide (*n* *=* 24), intravenous corticosteroid pulse (*n* *=* 9), and methotrexate (*n* *=* 3).^12^ At the time of recruitment, 41 patients (39%) were receiving prednisolone and 31 patients (29.5%) were on other oral immunosuppressants, such as azathioprine (*n* *=* 13), cyclophosphamide (*n* *=* 15), and methotrexate (*n* *=* 3).^12^

### Study design

At baseline, clinical history was assessed, and immunological investigations, such as DIF, serum autoantibodies, and peripheral blood CD19+CD27+ memory B cell by flow cytometry were performed.

Clinical and immunological follow-up were scheduled every 3 months for up to 12 months or until relapse, whichever occurred earlier. If relapse has not occurred by 12 months, some patients were followed up to a maximum of 24 months. After baseline, DIF was repeated only at the time of relapse or after 12 months. Those patients who had relapses after 12 months hence had biopsies for DIF at 3 time points: baseline, after 12 months, and at the time of relapse.^12^ According to an international consensus, relapse was defined as appearance of more than 3 new lesions in 1 month that did not subside spontaneously within 1 week.^15^

### Immunological investigations

Serum was separated from 7 mL blood in clot activator by centrifugation and aliquoted in 2 equal proportions and stored at −80 °C until assayed. The following 6 immunoserological parameters were determined from one part of serum: serum enzyme-linked immunosorbent assay (ELISA) reactivities for Dsg1 and Dsg3 (Euroimmun) and muscarinic3 acetylcholine receptor (M3 AchR) (Wuhan Fine Biotech). Conventional Dsg 1/3 ELISAs were performed after the manufacturer’s protocol. To detect conformational Ca^2+^ dependent epitopes on Dsg-1 and Dsg-3, ELISA plates were treated with 0.5 mmol EDTA for 30 minute at room temperature as described previously.^12^ After washing 4 times with ELISA wash buffers, the conventional assay protocol was followed. The difference between EDTA-untreated and EDTA-treated ELISA index represented the conformational ELISA index values. IgG1/IgG4 anti-Dsg3 ELISA was performed in samples with anti-Dsg 3 IgG ELISA values ≥5RU/mL using the conventional protocol^16^ replacing the anti-IgG detection antibody by peroxidase-conjugated sheep anti-human IgG1 (Binding Site, diluted 1:1,2500) and by peroxidase-conjugated mouse anti-human IgG4 (Southern Biotech, diluted 1:50,000).

Relevant pathogenic epitopes may cluster in Dsg1 and Dsg3's extracellular 1 (EC1) and EC2 domains, but current ELISA systems use the entire Dsg 1/3 ectodomain. Unlike IgG autoantibodies targeting other Dsg1 and Dsg3 epitopes, serum levels of these autoantibodies have correlated with disease activity.^17^^,^^18^ A biochip with EC1 and EC2 domains of Dsg3 as substrates was fabricated for detection of autoantibodies binding to these proteins. EC1-EC2, EC1-EC3, and EC1-EC4 of Dsg3 were recombinantly expressed on the surface of HEK293 cells as described.^19^ The EC Dsg3-expressing HEK293 cells were applied using the Biochip technology as previously reported with serum dilution of 1:10 and a fluorescein isothiocyanate (FITC)-labeled anti-human IgG/IgG4 detection antibody (Euroimmun).^20^^,^^21^ IgG reactivity against the EC domains 1, 2, 3, and 4 of Dsg3 was determined in 19 randomly selected patients, 9 of whom had disease relapse and others did not.

Three milliliters of EDTA blood were utilized for flow cytometry. After isolating lymphocytes through ficoll density gradient centrifugation, around 100,000 cells were suspended in polypropylene tubes. Memory B cells were labeled with FITC-conjugated anti-human CD19 and phycoerythrin (PE)-conjugated antihuman CD27 antibodies (BD Biosciences). After antibody excess removal, cells were analyzed on a flow cytometer. Isotype control antibodies were applied to stain the same cells in a separate tube as a negative control. In addition, unstained cells served as fluorescence minus one controls.

A 3 mm punch biopsy was collected from an area adjacent to the diagnostic DIF site. DIF was conducted using FITC-conjugated antibodies for IgA, IgM, IgG, and C3.

### Statistical analysis

Sample size was calculated for time-to-event analysis using a Cox proportional hazards model. We based our calculations on the following assumptions: a 5% significance level (α), a hazard ratio of 2, a SD of 1 for the key predictor variable (relapse), an R2 squared multiple-correlation coefficient of 0.2, an overall probability of an event (relapse) of 25% (Pr_E), and a withdrawal probability of 10% (Pr_w). These assumptions led to an initial requirement of 91 cases. However, to enhance the study’s power and the likelihood of observing relapses, 105 cases were included, and patients were followed for up to 24 months or until relapse, whichever occurred first.

For quantitative data, normality was assessed using Kolmogorov-Smirnov tests. Descriptive statistics were expressed as mean and SD or median and IQR. Comparisons between groups with and without relapse were performed using Student's t test for normally distributed data and the Mann-Whitney U test for skewed data. Qualitative or categorical data, such as DIF results, were analyzed using χ^2^ or Fisher’s exact (probability) test as appropriate. All tests were 2-sided with a significance level of *P* = .05.

## Results

The mean age of the whole cohort was 48.1 ± 14.5 years. Females to males ratio was 1:0.54. Ninety-three patients (88.5%) had PV and 12 patients (11.5%) PF. The patients were in remission for a mean duration of 14.7 ± 24.4 months at study inclusion.

### Baseline parameters among relapsed and nonrelapsed patients

Twenty-nine (27.62%) patients experienced a relapse. The median time interval between recruitment in study and occurrence of relapse was 6 months (3-9 months). Thirteen patients relapsed between 0-3 months, 7 patients between 3-6 months, and 3 patients each between 6-9 months and 9-12 months. One patient each relapsed between 12-15 months, 15-18 months, and 21-24 months. The differences in clinico-demographic and immunological parameters at baseline between Indian and European patients in this study have recently been reported.^12^

Clinical and immunological parameters at the time of study inclusion were compared between patients who maintained remission and those who relapsed. Relapsed and nonrelapsed patients were similar in terms of age, gender, type of disease (PV or PF), duration of disease, and duration of treatment received (Table I). Duration of remission at the time of study inclusion was significantly longer in relapsed patients (*P* = .01). In relapsed patients, pruritus was significantly more common compared with patients without relapse (*P* = .04).

**Table I:** Baseline demographic and clinical parameters

Parameters	Relapsed (*n* *=* 29)	Nonrelapsed (*n* *=* 76)	*P*
Age, mean ± SD	46.3 ± 17.1	48.7 ± 13.5	.44∗
Gender (male: female)	10:19	28:48:00	.82†
Disease, n (%)			.24†
Pemphigus vulgaris	24 (82.7%)	69 (90.7%)	
Pemphigus foliaceous	5 (17.3%)	7 (9.3%)	
Duration of disease in months, median (IQR)	39 (22-81)	48 (29-88)	.35‡
Duration of treatment received before achievement of remission in months, median (IQR)	6 (3-14)	10 (4-24)	.08‡
Interval between disease onset and initiation of treatment (in months), median (IQR)	3.0 (1.0-5.0)	2.5 (1.0-6.0)	.34‡
Duration of current remission in months, median (IQR)	9 (5-19)	5 (3-11)	.01‡
Pruritus (yes: no)	18:11	30:46:00	.04†
Average maximum body surface area involvement during preceding active disease, median (IQR)	1 (0-5)	1 (0-6)	.97‡
Oral mucosal involvement in previous disease activity, n (%)			
Yes	22 (75.9%)	63 (84%)	.34†
No	7 (24.1%)	12 (16%)	
Site of onset in previous activity, n (%)			.68†
Oral mucosa	21 (72.4%)	49 (65.3%)	
Skin	8 (27.6%)	25 (33.3%)	
Not sure	-	1 (1.3%)	
Total no. of relapses, median (IQR)	1 (0-2)	1 (0-2)	.46‡
Continent (India: Europe)	29:00	46:30	.00§

∗ Paired t test.

† χ^2^ test.

‡ Mann-Whitney U test.

§ Fisher’s exact (probability) test.

Frequency of elevated serum anti-Dsg 1 IgG (≥20 RU/mL) at baseline was significantly higher in the relapsed group (Table II). Baseline elevated serum anti-Dsg 1 IgG was recorded in 11 (37.9%) relapsed patients compared with 9 (11.8%) patients without relapse (*P* < .001). The mean pathogenic antibody index for anti-Dsg3 IgG by conformational ELISA was higher in the relapsed group (23.16, IQR: 1.07-23.26) compared with the nonrelapsed group (14.8, IQR: 6.46-61.62), but this difference was not statistically significant (*P* = .89). IgG1/IgG4 anti-Dsg3 ELISA reactivities were comparable between relapsed and nonrelapsed patients at baseline. The presence of Anti-Dsg3 EC1+EC2 antibodies was observed in 8/9 (88.8%) of the relapsed group, compared with 6/10 (60%) in the nonrelapsed group, with a *P*-value of 0.3, indicating no statistically significant difference between the 2 groups.

**Table II:** Comparison of baseline immunological parameters among relapsed and nonrelapsed patients

Parameters	Relapsed (*n* *=* 29)	Nonrelapsed (*n* *=* 76)	*P*
Antidesmoglein 1 levels (RU/mL)			< .001∗
<20	18 (62.1)	67 (88.2)	
≥20	11 (37.9)	9 (11.8)	
Antidesmoglein 3 levels (RU/mL)			.78∗
<20	14 (48.9)	39 (51.3)	
≥20	15 (51.7)	37 (48.7)	
Conformational antidesmoglein 3 antibody levels**,** median (IQR)	15.3 (0.54-126.4)	8.55 (0.29-105.29)	.69
Pathogenic antibody index (conventional minus conformational antidesmoglein 3), median (IQR)	23.16 (1.07-23.26)	14.8 (6.46-61.62)	.89
Anti-M3 acetylcholine receptor levels**,** median (IQR)	0 (0-3.38)	0 (0-4.2)	.68
CD19 positive cells (% of total lymphocytes)**,** median (IQR)	7.6 (4.6-12.6)	11.6 (7.2-17.4)	.041
CD27 positive cells (% of total lymphocytes), median (IQR)	2.9 (1.8-4.8)	3.6 (2.1-5.4)	.294
CD19 + CD27 + cells (% of total lymphocytes), median (IQR)	0.9 (0.4-2.8)	1.4 (0.3-5.1)	.449
IgG + by DIF	7 (24%)	25 (32.8%)	.321∗
C3 + by DIF	7 (24%)	12 (15.7%)	.368∗
Anti-Dsg3 IgG1 (*n* *=* 60), n (%)			
Negative	2 (12.5%)	7 (15.6%)	.76∗
Positive	13 (87.5%)	38 (84.4%)	
Anti-Dsg3 IgG4 (*n* *=* 60), n (%)			
Negative	3 (25%)	11 (24.4%)	.97∗
Positive	12 (75%)	34 (75.6%)	
Anti-Dsg3 EC1+EC2 (*n* *=* 19), n (%)			.3∗
Negative	8 (88.8%)	6 (60%)	
Positive	1 (11.1%)	4 (40%)	

*DIF*, Direct immunofluorescence.

∗ χ^2^ test; all other assessments, Wilcoxon rank-sum (Mann-Whitney) test.

Flow cytometric data for peripheral blood CD19+, CD27+, and CD19+CD27+ B cells at baseline was available for 90 patients. In the nonrelapsed patients, a significantly higher number of CD19+ B cells was seen at baseline, whereas no significant difference in CD27+ and CD19+CD27+ B-cell numbers was detected. The proportion of patients with positive DIF were comparable between the 2 groups. Mean serum levels of anti-M3AchR antibody was also comparable between the groups at baseline (relapsed, 23.7 ± 76.5 ng/mL; nonrelapsed, 36.7 ± 126.1 ng/ mL; *P* = .68).

### Parameters at the time of and immediately preceding the relapse

To determine the evolution of immunological parameters before the relapses, we assessed the immediately preceding immunological events. Among relapsed patients, the proportion with positive Dsg1 and Dsg3 ELISA (≥20 RU/mL) was significantly higher at the time of relapse compared with the timepoint immediately preceding the relapse (Table III). Proportion of DIF positivity was also significantly higher at the time of relapse compared with the previous time point when it was performed (Table III). When immunological parameters at the time of relapse were compared to all other observations in all patients during the course of the study, higher mean anti-Dsg 1 levels were noted at the time of relapse (Table IV). Because a significant difference in number of patients in the 2 groups (relapse visit, *n* *=* 29 vs all other visits, *n* *=* 432), a statistical test to determine the significance in difference was not employed. Both anti-Dsg1 and anti-Dsg3 levels were consistently elevated at all time points in patients with relapse (Supplementary Fig 1, available via Mendeley at https://data.mendeley.com/datasets/2h53y7xnwj/1).

**Table III:** Comparison of immunological parameters at the time of relapse versus immediately preceding the relapse

Parameters	Immediately preceding the relapse visit	At the time of relapse	*P*
(No. relapsed = 29)			
Antidesmoglein 1 (RU/mL)			.01∗
<20	20 (68.9%)	18 (62%)	
≥20	9 (31.1%)	11 (38%)	
Median (IQR) antidesmoglein 1 titers (RU/mL)	8.7 (0.13-88.8)	2.33 (0-34)	.446‡
Antidesmoglein 3 (RU/mL)			.01∗
<20	17 (58.6%)	14 (48.3%)	
≥20	12 (41.4%)	15 (51.7%)	
Median (IQR) antidesmoglein 3 titers (RU/mL)	27.3 (1.2-147.4)	2.9 (0-130.9)	.923‡
Median (IQR) conformational antidesmoglein 3 titers (RU/mL)	0.05 (0-61.7)	4.1 (0.05-105.5)	.58†
Median (IQR) anti-M3 acetylcholine receptor titers (ng/mL)	10.9 (0-32.4)	0 (0-3.39)	.66‡
CD19 positive cells (% of total lymphocytes)**,** median (IQR)	10.1 (4.4-15.3)	7.6 (4.6-12.6)	.23†
CD27 positive cells (% of total lymphocytes)**,** median (IQR)	2.7 (1.8-4.3)	2.9 (1.8-4.8)	.37†
CD19 + CD27 + cells (% of total lymphocytes)**,** median (IQR)	1 (0.6-1.5)	0.9 (0.4-2.8)	.92‡
IgG + by DIF	7 (25%)	24 (85.7%)	< .001§
C3 + by DIF	7 (25%)	20 (71.4%)	< .001§

*DIF*, Direct immunofluorescence.

∗ McNemar test.

† Paired t test.

‡ Wilcoxan signed-rank test.

§ χ^2^ test.

**Table IV:** Comparison of immunological parameters at the time of relapse to all other observations recorded during the study period

Parameters	At the time of relapse (*n* *=* 29)	All other observations in all patients
		(n = 432; 461-29 = 432)
Antidesmoglein 1 titers (RU/mL), median (IQR)	9 (0-89)	1.97 (0.39-7.47)
Antidesmoglein 3 titers (RU/mL), median (IQR)	27 (1-147)	15.13 (3.26-184.4)
Conformational antidesmoglein 3 titers (ng/mL), median (IQR)	4 (0-106)	9.65 (3.17-105.1)
Anti-M3 acetylcholine receptor titers, median (IQR)	0 (0-3)	4.95 (0-29.05)
CD19 positive cells (% of total lymphocytes), median (IQR)	8 (5-13)	10.35 (5.67-15.63)
CD27 positive cells (% of total lymphocytes), median (IQR)	3 (2-5)	3.4 (2.1-5.4)
CD19 + CD27 + cells (% of total lymphocytes), median (IQR)	1 (0-3)	1.2 (0.7-2.6)
IgG + by DIF	24 (85.7%)	55 (32.3%)∗
C3 + by DIF	20 (71.4%)	32 (18.8%)∗

Median IQR reported for not normally distributed data. *DIF*, Direct immunofluorescence.

∗ Total DIF observations: 199 (*n*_*1*_ = 29 for relapse, *n*_*2*_ = 170 for other observations.

## Discussion

Pemphigus follows a prolonged course with remissions and relapses. There is no standard guideline on how long treatment should be continued after remission is achieved and when to restart treatment based on immunological clues preceding impending relapse. In general, in patients with negative DIF and normal anti-Dsg IgG serum levels, most clinicians will favor tapering-off pemphigus treatments.^11^^,^^22^ In this prospective study, immunological parameters were periodically assessed to determine their use in the prediction of relapse among pemphigus patients. Presence of pruritus and longer duration of clinical remission at baseline were the clinical parameters associated with disease relapse.

High anti-Dsg1 serum levels at baseline were significantly linked to relapse. Relapse correlated with positive serum anti-Dsg1 IgG, anti-Dsg3 IgG, and positive DIF just before relapse. This implies that periodic assessments of anti-Dsg1 IgG, anti-Dsg3 IgG, and DIF can predict relapse in pemphigus. Thus, regular monitoring of anti-Dsg 1/3 serum levels by ELISA and performing DIF before discontinuing pemphigus medication is strongly recommended based on our findings. The recent European guidelines for PV/PF management only strongly recommend monitoring anti-Dsg1 levels.^13^

Previous studies with smaller samples identified pruritus as a pemphigus relapse predictor. In our study, patients experiencing relapse showed significantly higher baseline pruritus.^23^^,^^24^ In addition, extended remission at enrolment associated with an increased relapse likelihood (Table I). This hints at resurging B-cell activity and autoantibody production, leading to clinical relapse over time.

High baseline anti-Dsg1 levels were prevalent in our relapsed group, aligning with findings of Genovese et al.^9^ They reported that failing to achieve anti-Dsg1 IgG negativity at remission predicted relapse in patients with initial anti-Dsg1 and anti-Dsg3 positivity. Daneshpazhooh et al^10^ noted a significantly shorter relapse-free time in anti-Dsg 3-positive patients compared with anti-Dsg 3-negative patients. Positive DIF at baseline is another immunological parameter that has been associated with shorter duration of remission and higher likelihood of relapse.^10^^,^^24^ On the contrary, the proportion of DIF positivity was comparable between the groups at baseline in our study. However, more relapsed patients had DIF positivity with IgG and C3 at relapse compared with baseline (for those relapsing within 12 months) or at 12 months (for those relapsing between 12 months and the study’s end).

A statistically nonsignificant rise in mean anti-Dsg1 and Dsg3 serum levels was seen in this study at the time of relapse when compared with values immediately preceding the relapse. Similar observations were made by Abasq et al,^11^ who noted a correlation of anti-Dsg1 antibody ELISA values with cutaneous relapse. However, no correlation was observed between anti-Dsg3 ELISA values and the course of mucosal lesions.

In post-hoc analysis of a randomized controlled trial, Mignard et al^8^ found high baseline pemphigus disease area index and high levels of anti-Dsg1 and anti-Dsg3 IgG at month 3 to be significantly associated with relapse after rituximab. As none of the patients who received rituximab (n = 23) in this study experienced relapse, similar assessment was not possible.

The discordance between conventional anti-Dsg3 ELISA values and clinical disease activity as reported in some studies^11^ may be because of 2 reasons. Some antibodies detected by ELISA are nonpathogenic as they are directed against nonpathogenic epitopes of Dsg. Pathogenically relevant epitopes may cluster in the EC1 and EC2 domains of Dsg1 and Dsg3.^17^^,^^18^ The proportion of our patients with anti-Dsg3 EC1+EC2 reactivity was similar among relapsed and nonrelapsed patients. Secondly, IgG4 subclass antibodies are believed to be pathogenic and IgG1 are not.^25^ Positivity for anti-Dsg3 IgG4; however, was similar among our relapsed and nonrelapsed patients at baseline. Though hypothesized to be good predictors of relapse, conformational ELISA values representing pathogenic antibody index and anti-M3 AchR antibody were not found to be different between relapsing and non- relapsing patients.^26^^,^^27^

Strengths of our study include the prospective multicentre design, a relatively large number of patients, the long observation period, and multitude of immunological parameters assessed. The study was limited by the observation that all relapsed patients came from 1 center as intensively discussed elsewhere.^12^ We did not record whether relapsing PV cases were mucosal-dominant or mucocutaneous subtypes, which precluded a more detailed subgroup analysis of the predictive significance of antidesmoglein antibody titers for relapse.

## Conclusion

Pruritus and longer remission predict pemphigus relapse. Anti-Dsg1 IgG at remission onset indicates relapse risk. Positive anti-Dsg1 and anti-Dsg3, plus DIF after remission, predict relapse. This underscores regular monitoring of anti-Dsg 1 and 3 during the disease course, especially after remission. Our data suggest to perform a DIF before complete tapering off of immunosuppressives.

## Conflicts of interest

Dr De: investigator for Argenx, Beck: euroimmun, material for collaborative research project, Schmidt: scientific cooperation and patents with Euroimmun. Dr Vassileva: payment or honoraria for lectures, presentations, speakers bureaus, manuscript writing, or educational events—Pfizer, UCB, Novartis, Roche, Johnson & Johnson, Eli Lilly, Abbvie; participation on a Data Safety Monitoring Board or Advisory Board—Abbvie, Pfizer, Novartis. Dr Patsatsi: consulting—fees Abbvie, Argenx, Leo Pharma, Novartis, Genesis Pharma, Jannsen, Pfizer, UCB, Pharmaserv Lilly; payment or honoraria for lectures, presentations, speakers bureaus, manuscript writing or educational events—Abbvie, Leo Pharma, Novartis, Genesis Pharma, Jannsen, Pfizer, UCB, Pharmaserv Lilly; support for attending meetings or travel—Abbvie, Novartis, Genesis Pharma, Jannsen, Pfizer, UCB. The other authors declare no conflicts of interest.
